# Crystal Structure of Allophycocyanin from Marine Cyanobacterium *Phormidium* sp. A09DM

**DOI:** 10.1371/journal.pone.0124580

**Published:** 2015-04-29

**Authors:** Ravi Raghav Sonani, Gagan Deep Gupta, Datta Madamwar, Vinay Kumar

**Affiliations:** 1 BRD School of Biosciences, Vadtal Road, Satellite Campus, Sardar Patel University, Vallabh Vidyanagar, India; 2 Protein Crystallography Section, Solid State Physics Division, Bhabha Atomic Research Centre, Mumbai, India; NCI-Frederick, UNITED STATES

## Abstract

Isolated phycobilisome (PBS) sub-assemblies have been widely subjected to X-ray crystallography analysis to obtain greater insights into the structure-function relationship of this light harvesting complex. Allophycocyanin (APC) is the phycobiliprotein always found in the PBS core complex. Phycocyanobilin (PCB) chromophores, covalently bound to conserved Cys residues of α- and β- subunits of APC, are responsible for solar energy absorption from phycocyanin and for transfer to photosynthetic apparatus. In the known APC structures, heterodimers of α- and β- subunits (known as αβ monomers) assemble as trimer or hexamer. We here for the first time report the crystal structure of APC isolated from a marine cyanobacterium (*Phormidium* sp. A09DM). The crystal structure has been refined against all the observed data to the resolution of 2.51 Å to R_work_ (R_free_) of 0.158 (0.229) with good stereochemistry of the atomic model. The *Phormidium* protein exists as a trimer of αβ monomers in solution and in crystal lattice. The overall tertiary structures of α- and β- subunits, and trimeric quaternary fold of the *Phormidium* protein resemble the other known APC structures. Also, configuration and conformation of the two covalently bound PCB chromophores in the marine APC are same as those observed in fresh water cyanobacteria and marine red algae. More hydrophobic residues, however, constitute the environment of the chromophore bound to α-subunit of the *Phormidium* protein, owing mainly to amino acid substitutions in the marine protein.

## Introduction

Phycobiliproteins (PBPs) and linker proteins (LPs) are mutually arranged in cyanobacteria and red algae to form multi-molecular assemblies of around 4–8 MDa called phycobilisomes (PBS) [1–3]. Morphologically, PBS are composed of two major sub-structures with core situated on outer surface of thylakoid membrane and rods radiating out from core [4–6]. Rods contain phycocyanin (PC, absorption λ_max_ ~610–620 nm) and/or phycoerythrin (PE, absorption λ_max_ ~540–570 nm), and associated LPs. The core invariantly contains allophycocyanin (APC, absorption λ_max_ ~653 nm) and associated LPs [3]. Phycobilisomes harvest the sunlight and exhibit efficient energy transfer in the direction from PE → PC → APC → *chlorophyll* [7]. The energy absorbed by *chlorophyll* is used to split water molecules generating electrons and protons in the photosynthetic reaction centers. It has recently been shown that phycobilisomes supply energy to both the photosystems I and II [8].

Structural assemblies of different phycobiliproteins within PBS were found to be quite similar, despite divergence in the amino acid sequence [9]. The heterodimer (αβ monomer) of two homologous α- and β- subunits of PE, PC and APC proteins is the building block of PBS. Each subunit contains covalently attached non-cyclic tetrapyrrole chromophore(s) enabling the PBPs to absorb and emit light within specific wavelength range across solar spectrum. Three αβ monomers associate into the disc shaped trimers, which have often been observed to form hexamers forming the rods and core cylinders [9,10]. Linker peptides occupy the central cavity of 25 to 50 Å diameter within the rods/core cylinders and play central role in tethering up the PBPs together in a way that governs the expression of unique spectral properties of chromophores within PBS [11,12]. The energy absorbed by the chromophores of PC and PE is funneled through APC discs and linker proteins to *chlorophyll*. The unique red-shifted absorbances of APC chromophores and their fluorescence emission overlapping to that of *chlorophyll* has been found to play major connecting role in this light funneling phenomenon [13].

Allophycocyanin is bound to the same chromophore as that of phycocyanin, namely phycocyanobilin (PCB), and these interactions result in distinct 650 nm absorption band of APC. The red shift in the absorption of allophycocyanin bound PCB has been proposed to occur due to its surrounding protein micro-environment within the quaternary structure of the protein [14]. Also, configuration and conformation of the chromophores are thought to contribute in modulating the chromophore energies bound to different phycobiliproteins [3]. To elucidate the molecular interactions of chromophores and APC, a number of X-ray diffraction studies have been reported for diverse APC proteins isolated from fresh water cyanobacteria and marine red algae [15–18].

Potential applications of phototrophic cyanobacteria for the generation of renewable energy by optimizing their photosynthetic pathways have been discussed [19–20]. Clearly, the spectral range over which organism is able to capture sunlight and efficiency of transfer to the photosynthetic apparatus could play critical role in exploring its utility in capturing solar energy. It has also been established that PBS using cyanobacteria are more abundant in nutrient rich waters with high *chlorophyll* concentrations [21,22]. Intuitively, a marine cyanobacterium, adapted to low light, could be expected to be efficient in light capture and energy transfer to the downstream molecular machinery.

In the present paper, we report the first crystal structure of APC isolated from the marine cyanobacterium, *Phormidium* sp. A09DM (formerly known as *Lyngbya* sp. A09DM). The overall trimeric quaternary fold of the *Phormidium* protein resembles the other known APC structures from red algae and fresh water cyanobacteria. However, more hydrophobic residues in the microenvironment of the PCB chromophore bound to α-subunit of the marine cyanobacterium are observed in the crystal structure.

## Materials and Methods

No specific permissions were required for obtaining *Phormidium* sp. A09DM cyanobacterium. The bacterium was isolated from open rocky shores of Okha, Gujarat, India (22.3597° N, 69.5375° E) and was used as a source of APC protein. We also confirm that the field studies did not involve endangered or protected species.

### Cultivation of organism for APC production

*Phormidium* sp. A09DM, isolated from rocky shores of Okha, Gujarat, India, was used as a source of APC. Set of standard laboratory conditions described elsewhere [23–26] were used for the cultivation of *Phormidium* sp. A09DM. In brief, they were grown in artificial salt nutrient (ASN)—III medium under cool white light (36 W, 130 μmol photons m^-2^ s^-1^, 12:12 hours light: dark cycles) at 27 ± 2°C.

### Purification and characterization of *Phormidium* APC

The exponentially growing cyanobacterial cells (28 days after inoculation) were harvested by centrifugation at 3000 ×g for 15 min (Kubota 6500, Bunkyo-Ku, Tokyo, Japan) at 20°C. Allophycocyanin was isolated and purified from pelleted cell mass using the method described earlier [27,28] with slight modification. Briefly, pelleted cell mass was washed and re-suspended in five volumes of the extraction buffer (10 mM Tris-HCl, pH 8.1) for freeze-thaw cycles from -25°C to 4°C. The brick red supernatant, obtained after centrifugation, was subjected to 40% ammonium sulfate precipitation in the presence of 0.01% Triton X-100 to precipitate non-targeted proteins. Saturation of supernatant was further increased to 70% to pellet down the APC protein. Protein pellet was suspended in 10 mM Tris—HCl buffer (pH, 8.1) and APC was purified by passing through gel permeation column (350 × 10 mm) packed with Sephadex G-150 by using 10 mM Tris-HCl buffer (pH, 8.1) as mobile phase. Allophycocyanin rich fractions were pooled and loaded onto DEAE-cellulose anion exchanger (Column dimension: 40 × 10 mm) to achieve crystallization grade purity. Bound proteins were eluted with NaCl step gradient in 10 mM Tris-HCl buffer (pH, 8.1). Highly pure APC fractions were desalted and concentrated by ultra-filtration using a Macrosep (10 kDa MWCO centrifugal device, Pall Corporation). Purification was carried out at 4°C. Buffers used in the protocol were prepared in mili-Q water supplemented with 0.01% sodium azide.

Purified APC was assessed for purity, homogeneity, integrity and functionality by well-established standard biochemical methods [29–31]. Polyacrylamide gel electrophoresis (PAGE) was performed in non-denaturing as well as denaturing conditions as described by Singh et al. [29]. The resolved gels were stained according to Garfin [32]. Presence of APC was confirmed by bilin specific zinc-acetate staining [33], which appeared as orange fluorescence bands under UV trans-illumination (AlphaEase FC Imaging System, Alpha Innotech Corp., USA). The masses of APC component proteins were verified by MALDI-TOF mass spectrometry.

Spectroscopic properties of purified APC were investigated by UV-visible absorption and fluorescence emission spectroscopy as described previously [34]. Absorbance of the protein was recorded over 250–750 nm wavelength range on UV-Visible Spectrophotometer (Analytik Jena AG Specord 210, Germany) at 25°C. Protein purity was inferred from UV-visible spectrum using ‘purity ratio’ that was calculated from absorbance ratio, A_653_/A_280_. Protein concentration was estimated according to the method of Lowry et al. [35] with BSA used as the standard. Allophycocyanin content was estimated from UV—visible spectrum by using the equation of Bennet and Bogorad [36]. To adjudge the functional integrity, fluorescence emission spectrum of APC was traced upon excitation at 645 nm by fluorescence spectrophotometer (F-7000, Hitachi High Technologies, Japan).

The purified *Phormidium* APC protein was loaded onto the Superdex 200 10/300 GL column, pre-equilibrated with buffer containing 10 mM Tris-HCl (pH, 8.0) and 100 mM sodium chloride, for molecular weight determination. The Superdex 200 column was calibrated with gel filtration molecular weight markers (Carbonic anhydrase, 29 kDa; Ovalbumin, 44.3 kDa; Bovine serum albumin, 67 kDa; Apoferritin, 440 kDa; Bovine thyroglobulin, 669 kDa). The oligomeric status of the protein was estimated from the molecular weight calculated from the elution profiles of the APC protein and molecular weight markers.

### Sequence determination

Genes of allophycocyanin α- and β- subunits (*apcA* and *apcB*, respectively) were amplified in 30 μL PCR reactions consisting of 1X buffer (10 mM Tris pH 9.0, 50 mM KCl, 1.5 mM MgCl_2_, 0.1% Triton X-100), 0.33 mM each of dNTPs, ~100 ng of template DNA, 1.5 U of Taq DNA polymerase and 0.66 pmoles each of primers (Table 1). The primers used were designed on the basis of conserved nucleotide sequences of *apcA* and *apcB* genes available in UniProt database. Amplification program was set with 35 cycles of 1 min denaturation step (94°C), 1 min annealing step (55°C), and 1 min elongation step (72°C) using Biorad iCycler version 4.006 (Biorad, CA, USA). The 35 cycles were appended with initial denaturation step at 94°C of 5 min and a final extension step at 72°C for 20 min. The ~0.5 kb PCR products of *apcA* and *apcB* were sequenced by the automated DNA Analyzer 3730xl using BigDye Terminator v3.1 sequencing chemistry (Applied Biosystems, Foster City, CA). Partial gene sequences were submitted to NCBI GenBank with the accession nos. **LM993862, LM993863**. Partial amino acid sequences of the α- and β- subunits were deduced from these gene sequences (**GenBank accession nos., CDY72720, CDY72721**).

**Table 1:** List of designed primers for amplification of *apc*A and *apc*B genes.

Gene	Primer	Sequence (5’– 3’)
***apc*A (485 bp)**	Forward primer	ATGAGTATCGTCACTAAATCCATCG
	Reverse primer	TACTGCATTGCACCGACAAC
***apc*B (485 bp)**	Forward primer	ATGCAAGACGCAATTACTTCCG
	Reverse primer	TAGCTTAAGCCAGAGCAGATGTAG

### Crystallization of the protein and data collection statistics

Crystallization of *Phormidium* APC (4 mg/mL) was attempted using sitting—drop vapor diffusion method and formulated crystallization screens, including PEG, JCSG+, PACT and MBClass suites from Qiagen. The protein yielded crystals with a number of conditions containing PEG 6000 (16–20%) at pH of about 8. Crystals of different morphologies were observed to grow in the same wells of JCSG+ conditions containing additives like ZnCl_2_ and MgCl_2_. The diffraction experiments showed a strong tendency of twinning in some of these crystals, while other hexagonal plates diffracted poorly. Diffraction quality crystals of the protein were obtained by sitting-drop method using Intelli-well (ARI, Hampton) crystallization plates and JCSG+ crystallization condition containing 100 mM bicine (pH, 8.5) and 20% (w/v) PEG 6000 at 15°C. The crystals grew to the size of about 120x100x60 μm^3^ in 15 days. For diffraction experiments at 100 K, these crystals were flash cooled after immersing in 20% glycerol. These crystals belonged to space group *H* 32 and diffracted to about 2.5 Å resolution using fine-focus X-ray source (Microstar, Bruker) under cryo-conditions. The 3-dimensional diffraction intensity data from the crystals of *Phormidium* APC were acquired on an image plate detector (MARRESEARCH) using Cu Kα radiation with 1.0° oscillation per image. The data were processed using XDS/autoPROC [37].

### Structure solution and refinement

The initial phases for *Phormidium* APC crystals were obtained by the molecular replacement method using Phaser suite [38] and atomic coordinates of an αβ monomer of the red algae APC (PDB ID: 1KN1), without solvents and phycocyanobilin chromophores, as search model. The initial phases with the molecular replacement solution were reasonably accurate such that electron density for most of the known amino acids of α- and β- subunits (**GenBank accession nos., CDY72720, CDY72721**) could be identified in the electron density maps. The electron density could also be seen for the PCB chromophores (CYC) at lower contour levels. Only partial amino acid sequences of α (9–161) and β (1–155) subunits of *Phormidium* allophycocyanin were available from nucleotide sequencing experiments. Amino acid sequences for the N-terminal eight amino acids of the α-subunit and for the C-terminal six amino acids of the β-subunit were derived from the consensus sequence of allophycocyanin proteins available in NCBI. These residues and four corrections in the nucleotide derived amino acid sequence were subsequently validated in the refined structure. The individual atomic coordinates were refined by Phenix [39] with maximum likelihood target and with model building using COOT [40]. The covalent bonds between the Cys residues and CYC ligands were restrained to distance of 1.80Å in Phenix cycles of refinement. The progress in the refinement of the atomic model was monitored by R_work_ and R_free_. The atomic coordinates and structure factors have been deposited in the Protein Data Bank with the accession no. **4RMP**.

### Analysis for chromophore-protein interactions

The 3D coordinates of the trimer assembly of the known APC structures, available in PDB, were generated by PISA/COOT. The 2D projection diagrams of chromophore-protein interactions for the *Phormidium* APC protein and other known APC structures were generated from the 3D coordinates of trimer assembly for each of the protein using LigPlot+ software [41]. Default parameters (distance of 3.35Å for identifying H-bonds and maximum distance of 3.9 Å for non-bonded contacts) were used to portray H-bond interactions and hydrophobic contacts between the chromophores and protein amino acid residues. The identified protein contact residues for a chromophore were marked in the multiple sequence alignment of known structures achieved using Clustal Omega [42]. The differences in the microenvironments of PCB chromophores in allophycocyanin proteins from different sources were elucidated from this alignment. In parallel, multiple sequence alignment of APC proteins available in NCBI was also carried out using COBALT [43]. The top 100 sequences homologous to *Phormidium* APC were analyzed for conservation of identified protein contact residues.

## Results and Discussion

### Purification and characterization

**Fig 1:**
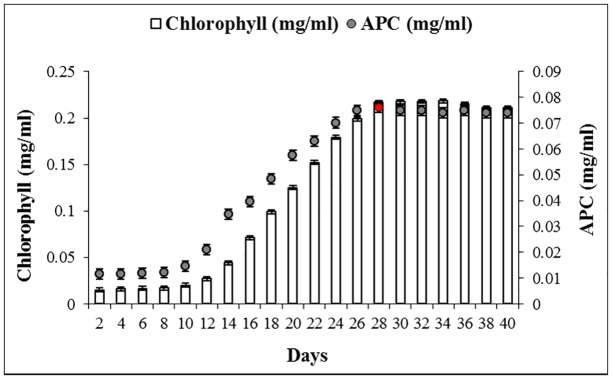
Growth pattern (in terms of chlorophyll, vertical bars) and APC production profile (filled circles) of *Phormidium* sp. A09DM, grown for 40 days. Allophycocyanin content was calculated using the Bennet and Bogorad [36] equations.

**Fig 2:**
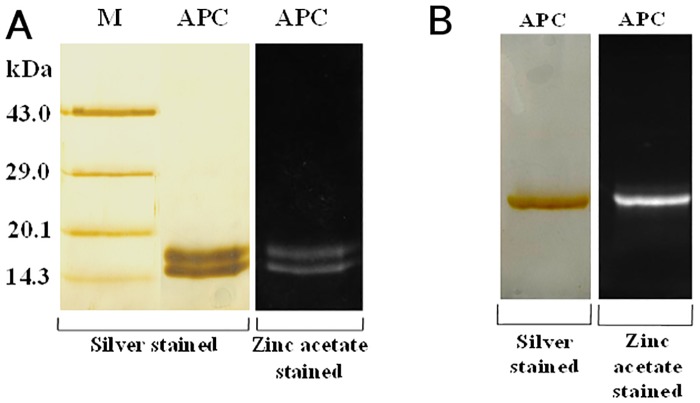
PAGE analysis of the purified allophycocyanin. **A**) Silver stained and zinc acetate stained 15% SDS-PAGE analyses of purified *Phormidium* APC. Protein molecular mass standard are shown in lane M. Only two bands were observed on silver stained and zinc acetate stained SDS-PAGE. These correspond to α- and β- subunits of the purified APC, suggesting also absence of linker peptide in purified APC complex. Nearly 10 μg of APC protein was loaded in each lane. **B**) Silver stained and zinc acetate stained 12% Native-PAGE of purified *Phormidium* APC further confirm homogeneity of the purified protein.

**Fig 3:**
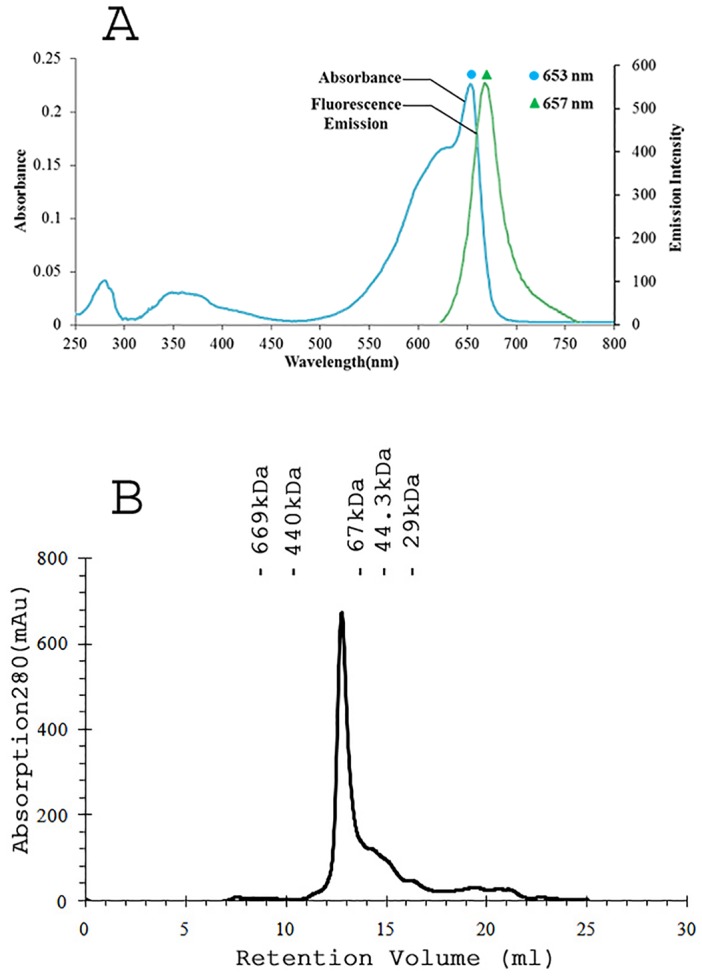
*Phormidium* APC exists as trimer of αβ monomers in solution. **A**) UV-visible absorption spectrum (cyan) of purified *Phormidium* APC showed major band at 653 nm that suggests formation of trimer in the solution. The fluorescence emission spectrum (green) of purified APC was measured upon excitation at 645 nm. **B**) The Superdex 200 gel-filtration profile of the *Phormidium* protein. The APC protein elutes at volume corresponding to an oligomer of 118 kDa. The column was calibrated with gel filtration molecular weight markers (Carbonic anhydrase, 29 kDa; Ovalbumin, 44.3 kDa; Bovine serum albumin, 67 kDa; Apoferritin, 440 kDa; Bovine thyroglobulin, 669 kDa). Elution volumes of the marker proteins are shown in the Figure.

Exponentially growing cell mass (28 days old) was taken for the extraction and purification of APC (Fig 1). Ice crystal formation during successive freeze (-25°C)—thaw (4°C) cycles ruptured the cell wall and facilitated complete extraction of intracellular proteins from cyanobacterial cell. Crude extract was subjected for APC separation using ammonium sulfate precipitation in the presence of Triton X-100. Separated APC was further purified by chromatography techniques. SDS-PAGE profile of purified APC showed only two bands, which corresponded to α- and β- subunits, and substantiated the purity of APC preparation. It also suggested the absence of any linker peptide in purified APC complex (Fig 2A). Emission of orange fluorescence by these bands on zinc acetate stained SDS-PAGE upon UV- illumination indicated the presence of chromophore(s) with both subunits of APC (Fig 2A). Purified APC showed single band in both silver as well as zinc acetate stained native-PAGE (Fig 2B). The functional integrity of purified APC was confirmed by the characteristic emission band observed at around 657 nm upon excitation at 645 nm under fluorescence spectroscope **(** Fig 3A). UV-visible absorbance spectrum of purified APC showing dominant (653 nm) and shoulder (620 nm) absorption peaks matched well with previously described APC absorption pattern [30,31]. APC specific peaks dominating over the protein specific peak (at 280 nm) in UV-visible spectrum again signified the absence of linker as well as other cellular protein (Fig 3A). Purity ratio, total APC content and total protein content (Table 2) collectively substantiated the success of purification protocol. Peaks at 18056 and 17987 Da were observed in MALDI-TOF experiments that were expected to be due to α- and β- subunits (S1 Fig). The molecular mass of the *Phormidium* APC oligomer was determined to be 118 kDa based on its elution profile on the molecular sieve column (Fig 3B). Given the observed mass of 36043 Da for an αβ monomer, the *Phormidium* protein is expected to form trimer in the buffer containing 10 mM Tris-HCl (pH, 8.0) and 100 mM sodium chloride.

**Table 2:** *Phormidium* APC protein purification progress and yield.

Purification step	Total protein content (mg)	APC content (mg)	APC (%)	Impurities (%)	Purity ratio, A_653_/A_280_	Yield (%)
**Crude extract**	88.76	6.55	7.38	92.62	0.09	100.00
**Ammonium sulfate precipitation**	8.20	5.31	64.75	35.25	2.08	81.20
**Chromatographic purification**	4.84	4.71	97.31	2.69	5.43	71.91

### Crystallographic analysis

The APC crystal belonged to space group *H* 32, with unit-cell parameters a = b = 101.2 Å, c = 193.0 Å. Data statistics are shown in Table 3. Based on determined molecular mass of αβ monomer (36043 Da) and the volume of the asymmetric unit, a Matthews parameter [44] of 2.64 Å^3^ Da^-1^ and a solvent content of 53.4% suggested the highest normalized probability (P_tot_ 1.0) for one αβ monomer in the asymmetric unit. The molecular replacement calculations yielded a unique solution with LLG value of 313 (TFZ, 15.9). The initial molecular replacement phases were accurate to show electron density for the known amino acids and covalently bound chromophores. A total of 14 amino acid residues, those were deduced from the consensus sequence, were also found to fit well in the electron density maps. Also, four corrections in the partial amino acid sequence (**GenBank accession nos., CDY72720, CDY72721)** were deduced based on refined atomic parameters, electron density maps, and conservation of amino acids in the multiple sequence alignment of APC orthologs. These were also confirmed from the nucleotide sequence chromatograms using different primers. The mis-annotation of 9-V**N**A-11 to 9-V**K**A-11, 48-E**R**I-50 to 48-E**C**I-50 and 144-E**D**A-146 to 144-E**N**A-146 of the α-subunit were mainly due to weak signals in the chromatogram. While mis-annotation of 34-G**E**L-36 to 34-G**K**L-36 of the β-subunit was due to overlapping strong signal in the chromatogram. The molecular masses of chromophore bound subunits with improved amino acid sequences were calculated to be 18064 and 18011 Da for α- and β- subunits, respectively. The theoretical molecular masses match well with molecular mass of 18056 and 17987 Da observed in MALDI-TOF analysis.

**Table 3:** Crystallographic data statistics for *Phormidium* APC.

Unit Cell	101.2, 101.2, 193.0 (Å), 90, 90, 120(°)
Space group	*H* 3 2
Matthews coefficient (Å^3^/Da)	2.64
Solvent content (%)^*a*^	53.4
Resolution limits (Å)	30–2.51 (2.64–2.51)^*b*^
Unique reflections	13362
Redundancy	10.8 (10.7) ^*b*^
Completeness (%)	99.9 (99.8) ^*b*^
R_merge_	0.109 (0.557) ^*b*^
Mean I/ mean σ(I)	22.8 (5.3) ^*b*^
**Refinement statistics**	
Resolution range (Å)	30–2.51
Wilson B (Å^2^)	30.6
Final R_work_/ R_free_	0.158/0.229
Ramachandaran plot^*c*^	98.7/1.0/0.3
Rotamer outliers (%)	0.4
Number of non-hydrogen atoms	2713
Root-mean-square deviation from ideality	
Bond lengths (Å)	0.008
Bond angles (°)	1.65

^a^. Estimated from Matthews coefficient with one (αβ) monomer per asymmetric unit.

^b^. Values in the highest resolution shell.

^c^. Percentage residues in the Ramachandran plot, Favored/Allowed/Outliers.

**Fig 4:**
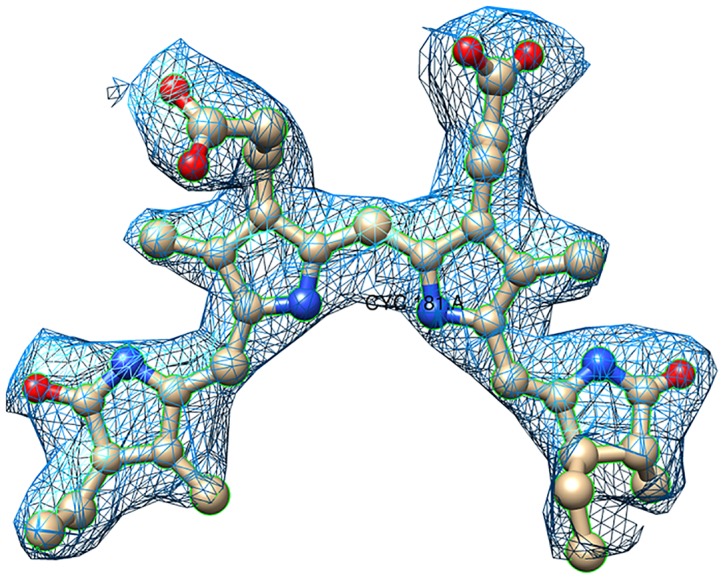
Representative electron density map. Shown is the fit of a PCB chromophore (ball-and-stick) in 2*Fo*—*Fc*, σ_A_-weighted electron-density map drawn at 1.2 σ contour level. The figure was prepared with Chimera suite [45].

The refined structural model of the APC protein has R_work_ (R_free_) of 0.16 (0.23) against all the observed data with F/σ(F) ≥ 0 (Table 3). The electron density for all the residues and both the chromophores is clearly defined (Fig 4). The evaluation using MOLPROBITY [46] revealed good stereochemistry of the structure, with nearly 98% residues in the most favored regions of the Ramachandran plot. Residue Thr-74 of the β-subunit lies close to disallowed region of the Ramachandran plot (with Φ, Ψ values of 78.3°, 137.3°) in all the known APC structures available in PDB. This residue resides on the loop region and its peptide amide accepts H-bond from the OB atom of the chromophore covalently linked with the α-subunit. Its main-chain carbonyl oxygen also accepts H-bonds from amide nitrogen atoms of Arg-77 and Tyr-88. It has been suggested that in the case of Thr-74 of β-subunit the specific requirements of the protein fold and ligand interaction override the usual Ramachandran constrains [47]. Interestingly also, the post-translationally modified Asn-71 (γ-*N*-methyasparagine, MEN) of the β-subunit also resides in the same loop. The methylation of this asparagine contributes to the efficiency of directional energy transfer in phycobilisomes [48]. The N-methyl group of MEN is clearly seen in the electron density maps and its side chain interacts with the chromophore of β-subunit (d_Oδ1-NC_ ~3Å).

### Structure analysis

The α- and β- subunits share nearly 35% sequence identity. The structures of α- and β- subunits are comprised of seven α-helices adopting globin-like fold (SCOP, 46457) and their folds are identical to that of other known APC structures. The subunit structures match with the rms deviation of 2.5 Å for 638 equivalent main chain atoms. The two subunits form a stable dimer (αβ monomer) with burial of nearly 5090 Å^2^ (~34%) of the solvent accessible area at the interface. The protein interfaces, surfaces and assemblies (PISA) service at EBI [49] estimated a gain of nearly 55.3 kcal/M in solvation free energy on the formation of αβ monomer. A large number of potential H-bonds and salt-bridges at the interface also contribute towards the stability of αβ monomer.

**Fig 5:**
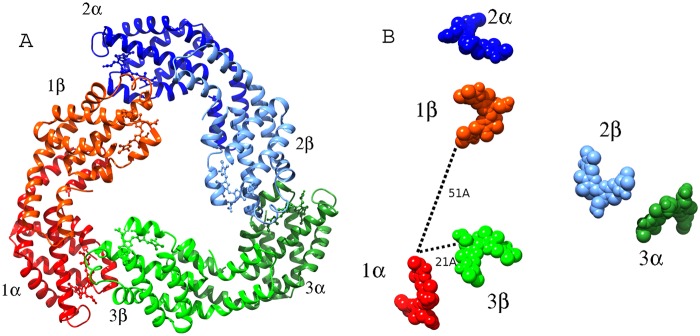
Trimer formation of αβ monomers of *Phormidium* APC results in close interactions of the chromophores of two subunits. **A)** Ribbon model of the trimer. Each αβ monomer is shown in two shades of the same color. The bound chromphores are shown as spheres. **B)** Chromophores (shown as spheres) are placed at 51Å apart (center-to-center distance) in an αβ monomer. Closer packing of the chromphores (d_center-to-center_ ~21Å) from different αβ monomers results due to oligomerization. The figure was prepared using Chimera suite.

Although only one αβ monomer is in the asymmetric unit, a trimer of the *Phormidium* protein exists in the crystal structure. Also the formation of trimer in solution is expected from the observation of the prominent 653 nm absorbance band and APC elution profile on the molecular sieve column as the protein maintained in low salt buffer (10 mM Tris-HCl and 100 mM sodium chloride) migrates as 118 kDa oligomer on the molecular sieve column. It has been recently suggested that hexamers are intrinsically less stable than trimers and are easily disrupted by small changes in the structure in solution lacking stabilizing phosphate [18]. The trimer in the crystals is generated by the crystallographic symmetry. Three αβ monomers of APC align side-by-side to form a hollow disc (Fig 5A) with an estimated gain of nearly 211 kcal/M of solvation free energy on trimer formation. The trimeric quaternary fold of *Phormidium* protein resembles the trimers of APC observed from fresh water cyanobacteria and red algal species.

Two trimers interact face-to-face to form hexamers in the crystals of APC from *P*. *yezoensis* and *G*. *violaceus* (PDB IDs: 1KN1 and 2VJT), and assembly of such hexamers into core cylinders is expected to be present in active phycobilisomes. However, *Phormidium* APC does not form extended assembly in the crystals. Also, packing of trimers in the crystal lattice of *Phormidium* APC differs from the APC structures of *S*. *platensis* (PDB ID: 1ALL), *T*. *vulcanus* (PDB ID: 3DBJ) and *S*. *elongatus* (PDB ID: 4F0U). Its C-termini helices from three α-chains of a trimer in a layer interact with three different laterally displaced trimers in another layer. A bilayer of laterally displaced trimers further forms loose association with other bilayers through their respective β-subunits. The packing of trimers in the structure of *Phormidium* APC thus matches with the interaction of trimers observed in the crystal structure of *T*. *elongatus* APC (PDB ID: 2V8A; [47]) resolved at 3.5Å resolution. It is interesting that crystals of *Phormidium* and *T*. *elongatus* APC proteins were obtained under totally different conditions. Crystals of *T*. *elongates* APC were obtained from 1 M ammonium sulphate, while PEG 6000 was used for crystallization of the *Phormidium* protein.

**Fig 6:**
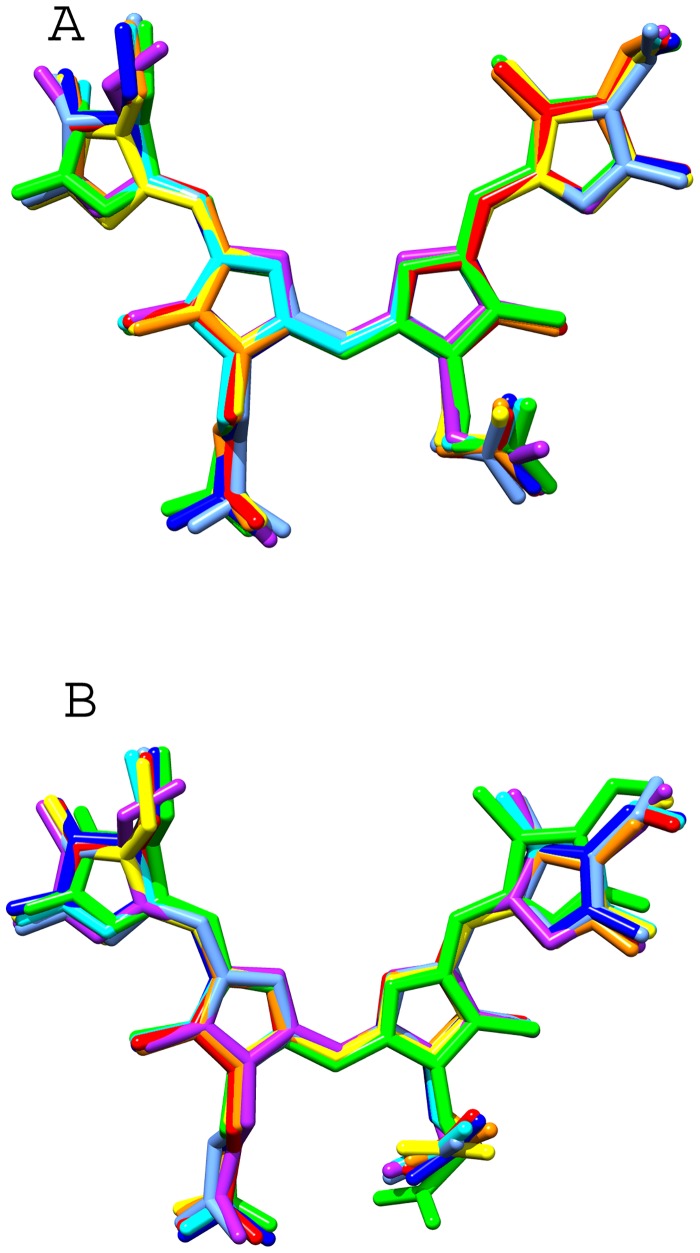
Configuration and conformation of the chromophores in the known APC structures. The atomic coordinates of PCB chromophores in the known APC structures (PDB IDs: 1ALL, 1KN1, 1B33, 2V8A, 2VJT, 3DBJ and 4F0U) were superposed onto the PCB chromophore of *Phormidium* APC by Chimera suite. **A**) Superposed chromophores bound to α-subunits **B**) Superposed chromophores bound to β-subunits.

**Fig 7:**
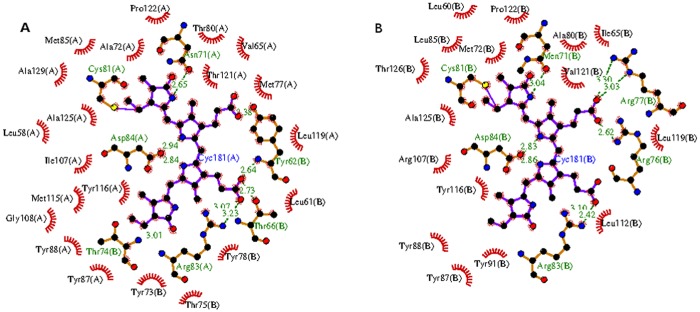
A LigPlus schematic 2D representation of the chromophore-protein interactions. Residues from α-subunit (shown as XXXnnn(A)) and from β-subunit (shown as XXXnnn(B)) within a distance of 3.9Å from the chromophore atoms are displayed. Non-chromophore residues involved in hydrophobic contact(s) are shown with residue labels. Side chains of residues forming covalent and H-bonds with the chromophore atoms are shown as sticks. H-bonds are shown in green dashed lines. Chromophore bonds are shown in purple. **A)** Microenvironment around the α-chromophore (labeled Cyc181(A) in the figure). **B**) Microenvironment around chromophore (Cyc181(B)) covalently bound to Cys-81 of β-subunit. The figure was prepared using LigPlus suite [41].

**Fig 8:**
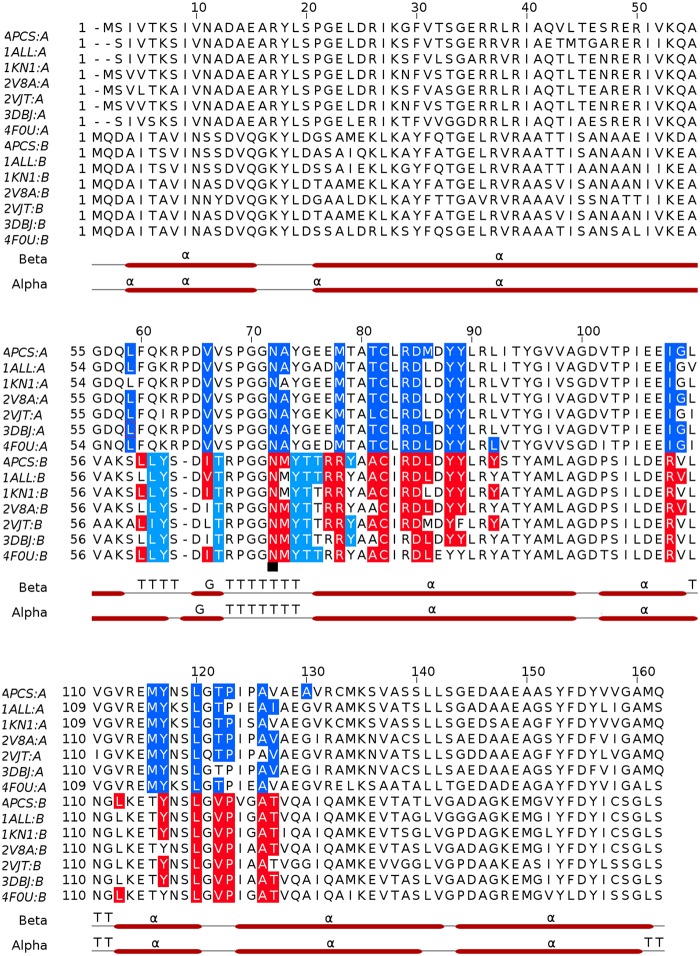
Multiple sequence alignment of α (A) and β (B) subunits of APC orthologs, and microenvironments of PCB chromophores in the known APC structures. APCS, *Phormidium*; 1ALL, *Spirulina platensis*; 1KN1, *Porphyra yezoensis*; 2V8A, *Thermosynechococcus Elongatus*; 2VJT, *Gloeobacter Violaceus*; 3DBJ, *Thermosynechococcus vulcanus*; 4F0U, *Synechococcus elongatus* PCC 7942. Multiple sequence alignment was achieved with Clustal Omega and residues which constitute microenvironments of PCB chromophores were detected using LigPlus suite. Blue shaded residues of α-subunit and cyan shaded residues of β-subunit are within non-bonded distance of 3.9Å from the chromophore atoms covalently linked to α-subunit. The β-subunit residues shaded in red are within non-bonded distance of 3.9Å from any of the chromophore atom covalently linked to β-subunit. Secondary structure of the Phormidium APC, as estimated using STRIDE (http://webclu.bio.wzw.tum.de/stride/; [51]), is also shown (α, α-helices; G, 3_10_ helices; T, Turns). The figure was prepared using Jalview [52]. The γ-*N*-methylasparagine residue of the β-subunit is marked with a black square.

One PCB chromophore is covalently bonded to the conserved Cys-81 of α-subunit (α-chromophore) and other chromophore is covalently linked to conserved Cys-81 of β-subunit. The two chromophores are separated by a distance of about 51Å (center-to-center) in an αβ monomer. The configuration and conformation of PCB chromophores in the two subunits of *Phormidium* APC do not differ from each other, as well as from the other known structures of APC from different species (Fig 6). However, microenvironments around chromophores differ markedly in α- and β-subunits (Fig 7). Trimer formation packs the amino acid residues of β-subunit closer to α-chromophore, while atoms of β-subunit only interact with its bound chromophore. The β-subunit contributes three Tyr, three Thr and one Leu residues to the microenvironment of the α-chromophore. Of these, Tyr-62 and Thr-66 form hydrogen bonds with the chromophore atoms (Fig 7). The bathochromic shift observed in APC upon trimerization has been attributed due to the coupling of the hydrophobicity of the α-chain chromophore environment and charged residues contributed by the β-chain [50]. The chromophores of α- and β- subunits in a trimer also interact with each other at shorter distance of about 10.5Å (center-to-center distance of ~21Å) (Fig 5B). Detailed analyses of the chromophore-protein interactions observed in the *Phormidium* marine APC, and other fresh water cyanobacteria and marine red algae suggest that hydrophobic contacts of the α-chromophore with Thr-75 and Tyr-78 residues of β-subunit and with Met-85 and Ala-129 of α-subunit are not conserved in all the known APC structures (Fig 8). These amino acid residues approach the α-chromophore of *Phormidium* APC within a distance of 3.9Å. Thr-75 and Tyr-78 residues are conserved in the known APC structures, while Met-85 and Ala-129 of the *Phormidium* protein are mostly substituted by Leu and Gly in other APC orthologs. The S^δ^ atom of Met-85 is placed at about 4.3Å from the S^γ^ atom of Cys-81 that forms a covalent bond with the α-chromophore. The C^β^ atom of Ala-129 interacts with the chromophore directly, and this interaction is not found in other known APC structures due to presence of Gly residue at the equivalent position in the alignment.

The protein sequence database search reveals that Met-85 is conserved in *Synechococcus* and *Cyanothece* marine cyanobacteria as well, whereas position 129 is occupied by Ala/Ser residues. Interaction between the α-chromophore and 129^th^ residue could be expected to be present in both the marine cyanobacteria. A database resource for marine cyanobacterial sequences is not presently available. We looked at the APC homologous sequences available at NCBI. Using the *Phormidium* APC as query sequence, top 100 most closely related sequences to *Phormidium* APC were aligned using COBALT. We observed that Met/Leu and Gly/Ala/Ser are the only residues found at positions 85 and 129 of α-subunit, respectively. Also, several species carrying Met and Ala/Ser at these positions were identified. These species are found both in fresh water (**F**) and marine water niches (**M**), and include *Nodosilinea nodulosa* (**F**), *Leptolyngbya* sp. Heron Island J (**M**), *Leptolyngbya* sp. PCC 7375 (**M**), *Synechococcus* sp. PCC 7335 (**M**), *Gloeocapsa* sp. PCC 7428 (**F**), *Synechococcus* sp. JA-3-3Ab (**F**), *Halothece* sp. PCC 7418, *Gloeocapsa* sp. PCC 73106 (**F**), *Synechococcus* sp. JA-2-3B'a(2–13) (**F**), *Dactylococcopsis salina* (**M**), *Arthrospira jenneri fb* (**F**), *Stanieria cyanosphaera* (**F**), *Pleurocapsa* sp. PCC 7319 (**M**), *Spirulina subsalsa* (**F, M**), *Rubidibacter lacunae* (**M**), *Synechococcus* sp. PCC 7336 (**M**), *Mastigocoleus testarum* (**M**), *Synechococcus sp*. *NKBG15041c* (**M**), *Synechococcus* sp. PCC 7002 (**M**), *Cyanobacterium stanieri* (**F**), *Cyanothece* sp. PCC 7424 (**F**), *filamentous cyanobacterium ESFC-1* (**M**) and *Cyanothece* (**F**). Clearly structural information on phycobiliproteins from organisms adapted to different sunlight niches shall be required to elucidate if the chromophore microenvironment alone affects the spectral properties and energy transfer efficiency of APC in cyanobacteria, and if microenvironment could be targeted for engineering energy capture and energy transfer efficiency.

## Conclusions

We have resolved the crystal structure of APC of marine cyanobacterium, *Phormidium* sp. A09DM. The protein is observed to exist as a trimer both in solution and in crystals. The overall tertiary structures of α- and β- subunits, trimeric quaternary fold, and configuration and conformation of the chromophores of the marine protein resemble the other known APC structures from red algae and fresh water cyanobacteria. However, microenvironment of the PCB chromophore bound to α-subunit is enriched by hydrophobic residues, owing to the presence of Met-85 and Ala-129 residues, in the *Phormidium* APC protein.

## Supporting Information

S1 Fig MALDI-TOF spectrum of the Phormidium APC. Two overlapping peaks were resolved in the expanded m/z scale and these showed masses of the two APC subunits to be 17988.3 and 18055.6 Da.
(PDF)
