# Supplementary material for: Crystal Structure of Allophycocyanin from Marine Cyanobacterium Phormidium sp. A09DM
Source: PLoS One. 2015 Apr 29;10(4):e0124580. doi: 10.1371/journal.pone.0124580 (PMC4414346; doi:10.1371/journal.pone.0124580)
Supplement: S1 Fig — Two overlapping peaks were resolved in the expanded m/z scale and these showed masses of the two APC subunits to be 17988.3 and 18055.6 Da. (PDF) [file pone.0124580.s001.pdf]

Supporting Information:

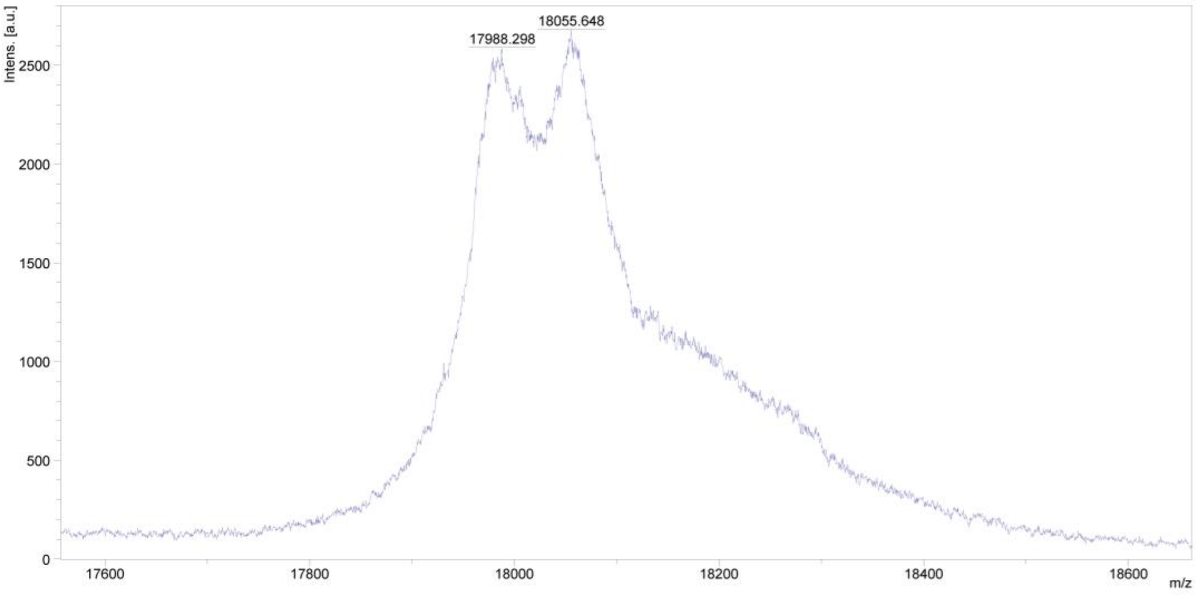

S1 Fig. MALDI-TOF spectrum of the Phormidium APC. Two overlapping peaks were resolved in the expanded m/z scale and these showed masses of the two APC subunits to be 17988.3 and 18055.6 Da.
